# Triggers of Guillain–Barré Syndrome: *Campylobacter jejuni* Predominates

**DOI:** 10.3390/ijms232214222

**Published:** 2022-11-17

**Authors:** Josef Finsterer

**Affiliations:** Neurology & Neurophysiology Center, 1180 Vienna, Austria; fifigs1@yahoo.de; Tel.: +43-1-5861075; Fax: +43-1-5861075

**Keywords:** polyradiculitis, Guillain–Barré syndrome, campylobacter jejuni, nerve roots, demyelination

## Abstract

Guillain–Barré syndrome (GBS) is a rare immune-mediated acute polyradiculo-neuropathy that typically develops after a previous gastrointestinal or respiratory infection. This narrative overview aims to summarise and discuss current knowledge and previous evidence regarding triggers and pathophysiology of GBS. A systematic search of the literature was carried out using suitable search terms. The most common subtypes of GBS are acute inflammatory demyelinating polyneuropathy (AIDP) and acute motor axonal neuropathy (AMAN). The most common triggers of GBS, in three quarters of cases, are previous infections. The most common infectious agents that cause GBS include *Campylobacter jejuni (C. jejuni)*, *Mycoplasma pneumoniae*, and cytomegalovirus. *C. jejuni* is responsible for about a third of GBS cases. GBS due to *C. jejuni* is usually more severe than that due to other causes. Clinical presentation of GBS is highly dependent on the structure of pathogenic lipo-oligosaccharides (LOS) that trigger the innate immune system via Toll-like-receptor (TLR)-4 signalling. AIDP is due to demyelination, whereas in AMAN, structures of the axolemma are affected in the nodal or inter-nodal space. In conclusion, GBS is a neuro-immunological disorder caused by autoantibodies against components of the myelin sheath or axolemma. Molecular mimicry between surface structures of pathogens and components of myelin or the axon is one scenario that may explain the pathophysiology of GBS.

## 1. Introduction

Guillain–Barré syndrome (GBS) is a rare immune-mediated acute polyradiculo-neuropathy that typically develops after previous gastrointestinal or respiratory infection, which is why it is also referred to as post-infectious polyneuropathy [1,2]. It can also be triggered by vaccinations and occurs in the paediatric and adult population. GBS has a broad clinical spectrum but most commonly manifests with progressive weakness of the limb, axial, facial, or respiratory muscles and with or without sensory or autonomic impairment [1,2]. Various infections agents, vaccinations, and other conditions have been identified as triggers of GBS, but a comprehensive summary of these causes is not available. This narrative overview aims to summarise and discuss current knowledge and previous evidence about common and rare conditions known to be associated with occurrence of GBS.

## 2. Results

### 2.1. Clinical Presentation of GBS

Clinical presentations of GBS are highly variable.

#### 2.1.1. GBS Subtypes

Different subtypes of GBS are defined according to clinical presentation and findings from nerve conduction studies (NCSs) (motor, sensory, axonal, demyelinating lesion). The most common of these subtypes, particularly in the western hemisphere, is acute inflammatory demyelinating polyneuropathy (AIDP). The most common subtypes in the eastern hemisphere are acute motor axonal neuropathy (AMAN) and acute motor and sensory axonal neuropathy (AMSAN). Other subtypes include Miller–Fisher syndrome (MFS) (ophthalmoparesis, ataxia, absent tendon reflexes), flail arm syndrome, flail leg syndrome, the pharyngo-cervico-brachial (PCB, axonal) subtype, mononeuritis cranialis (MNC), polyneuritis cranialis (PNC), acute bulbar palsy (ABP), and brainstem Bickerstaff encephalitis (BBE) [1,2].

#### 2.1.2. Neurological Exam

The clinical presentation of GBS is diverse, depending on the GBS subtype. In most cases, GBS presents with progressive ascending muscle weakness of the limbs with or without involvement of sensory or autonomic fibers. Occasionally, axial, facial, or respiratory muscles are also affected. Tendon reflexes are usually reduced, but hyperreflexia has occasionally been reported [3]. Hyperreflexia is explained by immune-mediated damage and dysfunction of inter-neuronal inhibitory circuits in the anterior horn [3].

#### 2.1.3. Cerebrospinal Fluid Investigations

Cerebrospinal fluid (CSF) investigations may show “dissociation cyto-albuminique”: a hallmark of GBS. CSF glucose and lactate are usually normal, and oligoclonal bands are negative. CSF tests can be normal at onset; therefore, normal findings in CSF investigations do not rule out GBS.

#### 2.1.4. Electrophysiological Studies

Application of NCSs may show increased distal latencies of motor nerves, reduced nerve conduction velocities (NCVs) of motor or sensory nerves, or reduced amplitudes of compound muscle action potentials (CMAPs) or sensory nerve action potentials (SNAPs) [4]. When sensory fibers are affected, somatosensory-evoked potentials (SSEPs) can show delayed cortical responses. NCSs can be normal at onset.

#### 2.1.5. Imaging

T1-weighted MRI with contrast medium of the cervical or lumbar spine can show thickening and enhancement of nerve roots or the cauda equine [5]. T1-weighted MRI of the brain can show thickening or enhancement of cranial nerve roots [5]. In case of brainstem involvement, T1-weighted magnetic resonance imaging (MRI) can show hyperintensity of the pons and bulb [6].

#### 2.1.6. Ganglioside Antibodies

Ganglioside antibodies are directed against gangliosides (sialic-acid-containing glycolipids) that are predominantly located at the nodes of Ranvier and at nerve terminals [2]. GM1-antibodies are generally elevated in 50–60% of AMAN cases but only in 7–35% of AIDP cases. GQ1b antibodies are elevated in 100% of MFS and BBE cases (GQ1b antibody syndrome). Antibodies against GQ2b, GM2, GM3, GM4, anti-titin, or anti-sulfatide are rarely increased. According to a study of 312 GBS patients from Bangladesh, serum GM1 antibodies were elevated in up to 60% of patients with the AMAN subtype and in up to one third of patients with the AIDP subtype [7]. Antibodies in *Mycoplasma-pneumoniae*-related AIDP are directed against galactocerebroside [2].

#### 2.1.7. Disease Course

The disease course is usually monophasic but can undulate. In up to one third of cases, respiratory muscles are involved, necessitating mechanical ventilation in up to one quarter of cases.

#### 2.1.8. Diagnosis

There are no mandatory criteria for diagnosing GBS, but several approaches have been taken to provide efficient diagnostic criteria. These approaches led to the development of the Asbury and Cornblath criteria [8], the Ashford criteria, the Haddon criteria, the Devigli or Besta criteria, and the Brighton criteria. The Brighton criteria, published in 2011, are the most widely used. They are based on the assessment of seven items (absence of alternative causes of weakness, reduced tendon reflexes of weak muscles, monophasic course, bilateral flaccid weakness, CSF cell count < 50 cells/microL, elevated CSF protein, NCSs consistent with a subtype). Differential diagnoses that must be ruled out before diagnosis of GBS include CIDP, which is preceded by an infection in only 10% of cases [9]; nodopathies (antibodies against contactin-1, contactin-associated protein-1, neurofascin-155, or pan-neurofascin are present); spinal muscular atrophy; amyotrophic lateral sclerosis; sensori-motor polyneuropathy; neuroborreliosis; myasthenia; and critical illness neuropathy.

### 2.2. Epidemiology of GBS

The incidence of GBS is reported as 1–2/100,000/y [10]. It is estimated that about 100,000 new cases are diagnosed worldwide each year. Males are more frequently affected than females [10]. There is a 20% increase in incidence for every 10 year increase in age [2]. About one third of patients require ventilatory support [2]. Mortality rate is 3–10%. In a study of 3486 GBS patients, the annual incidence was 1–1.2/100,000/y during the years 2009–2015 [11]. In this study, GBS incidence was higher in males (1.2/100,000) than in females (0.9/100,000) and increased with age, from 0.4/100,000 in persons 0–17 years old to 2.1/100,000 in persons ≥ 65 years old [11].

### 2.3. Triggers of GBS

#### 2.3.1. Infections

Infections are the most common triggers of GBS, preceding GBS in 75% of cases [2]. Bacteria and viruses have been implicated in the pathogenesis of GBS. In a study by Jacobs et al. from 1998, GBS was accordingly defined as an acute generalised poly-radiculo-neuropathy that is preceded by a symptomatic infection, such as *Campylobacter jejuni (C. jejuni)*, Epstein–Barr virus (EBV), cytomegalovirus (CMV), or influenza in about two thirds of cases [12]. By far, the most common bacterial agents that trigger GBS are *C. jejuni* and *Mycoplasma pneumoniae* [13]. The most common viruses that cause GBS are CMV, Zika, and dengue [14]. An analysis of the first 1000 patients enrolled in the International GBS Outcome Study (IGOS), for which 768 biosamples were available, showed that GBS was caused by *C. jejuni* in 30%, *M. pneumoniae* in 10%, CMV in 4%, hepatitis-E in 3%, and EBV in 1% [13]. Six percent of GBS patients had more than one previous infection [13]. Symptoms of previous infections were reported in 72%, and this proportion did not differ significantly between those who tested positive or negative for a recent infection [13]. Proportions of infections were similar across continents [13]. The sensorimotor variant and the AIDP subtype were the most common subtypes across all infection groups, with proportions that were significantly higher in patients with CMV and significantly lower in patients with *C. jejuni* infection [13]. GBS with a previous *C. jejuni* infection was more severe and required a longer time to regain the ability to walk independently [13]. The purely motor variant and the AMAN subtype were more common in Asian than in American or European *C. jejuni-*positive patients [13]. Time to nadir was longer in CMV as compared with GBS caused by other infections [13]. About half of the patients had cranial nerve involvement and 25% autonomic involvement [13]. In a study of 150 Chinese patients with GBS, the disease was preceded by infections with *C. jejuni* (27%), influenza-A (17%), influenza-B (16%), hepatitis-A (5%), dengue virus (3%), CMV (3%), EBV (3%), *M. pneumoniae* (2%), HSV (2%), VZV (1%), and rubella virus (1%) [15]. Serology for infections with hepatitis E virus, *Haemophilus influenzae*, and Zika virus was negative [15].

Bacteria

*Campylobacter jejuni*

Characteristics of *C. jejuni*

*C. jejuni* is a microaerophilic, oxygen-sensitive, gram-negative, and non-spore-forming bacterium of the genus *Campylobacter* and a common cause of gastroenteritis in humans [16]. The genome of this species was fully sequenced in 2005. *C. jejuni* is unusual for an intestinal pathogen in its ability to coat (envelop) its surface with a polysaccharide capsule (CPS) [17]. CPSs vary in sugar composition and linkage, particularly those involving heptoses of unusual configuration and O-methyl phosphoramidate linkages [17]. CPSs are sialylated lipo-oligosaccharides (LOSs) divided into four groups (1–4) and 23 classes (A to W) [18]. Structural diversity is consistent, with CPS as the major sero-determinant of the Penner serotyping scheme, of which there are 47 *C. jejuni* serotypes [17]. Different strains (Penner serotypes) originating from hosts such as cattle or poultry can be distinguished by LOS classification and multilocus sequence typing (MLST) [19].

Transmission to humans

Transmission of *C. jejuni* to humans is usually through food consumption: particularly consumption of raw or undercooked poultry meat; unpasteurised, contaminated milk; or water-based environmental sources [20]. After uptake, *C. jejuni* colonises the distal ileum and colon [20].

Diseases and complications caused by *C. jejuni*

*C. jejuni* can cause various human diseases. The most common human disease caused by *C. jejuni* is foodborne enteritis. *C. jejuni* is the leading bacterial cause of foodborne gastroenteritis in developed countries [21]. *Campylobacter* spp. are the leading cause of bacterium-derived gastroenteritis worldwide, affecting about 100 million individuals annually [22]. In contrast to other bacterial pathogens of the gastrointestinal tract, Campylobacter spp. lack many of the classic virulence factors often associated with the ability to cause disease in humans, including a range of canonical secretory systems and toxins [22]. In Germany, campylobacteriosis has increased the most in the warm season and has now reached up to 70,000 reported cases per year [23]. The most common species of campylobacteria that cause intestinal infections are *C. jejuni*, *C. coli*, and *C. upsaliensis* [23]. Enteropathogenic panels can detect campylobacteria quickly and reliably [23]. Infection with *C. jejuni* is also considered the most common cause of GBS [24]. Other complications of *C. jejuni* infections are irritable bowel syndrome [25], reactive arthritis, Reiter syndrome (arthritis, urethritis, iritis), spinal abscesses [26], and Achilles enthesopathy [27].

Pathophysiology

Importance of Lipo-Oligosaccharides

Post-infectious morbidity of human *C. jejuni* infection is highly dependent on the structure of the pathogenic LOS, which trigger the innate immune system via Toll-like-receptor (TLR)-4 signalling [28]. Toll-like receptors (TLRs) recognise distinct pathogen-associated molecular patterns. They participate in the first line of defence against invading pathogens and play a significant role in inflammation, immune-cell regulation, survival, and proliferation. To date, 11 members of the TLR family have been identified, of which TLR1, TLR2, TLR4, TLR5, TLR6, and TLR11 are located on the cell surface and TLR3, TLR7, TLR8, and TLR9 are localised to the endosomal/lysosomal compartment. Development of GBS through *C. jejuni* is explained by “molecular mimicry” between surface LOS antigens and ganglioside antigens (ceramides) on the surface of myelin sheaths or the axolemma. LOSs are an integral part of the *C. jejuni* cell membrane, with a structure of core oligosaccharides forming inner and outer core regions and a lipid-A moiety [18]. Outer membrane LOSs are also involved in processes such as colonisation, survival, inflammation, and immune evasion [29].

Clinical and serologic data support a model in which LOSs of specific *C. jejuni* strains elicit antibodies that recognise both bacterial molecules and gangliosides. They also elicit recognition of the latter biomolecules, which are abundantly expressed in the nervous system, where they are involved in neuro-transmission and cause neurological dysfunction [16]. LOSs of *C. jejuni* often produce structures that mimic the oligosaccharide moieties of GM1a and GD1a [16]. In addition, strains that express GD3, GM2, GM3, and GT1a mimics have been isolated [16]. Whether the presence of antibodies against *Helicobacter pylori* contributes to the pathophysiology of GBS remains unclear, but there is evidence that *Helicobacter pylori* antibodies are more common in GBS patients than in controls [30].

The Classical Immune Mechanism

Exposure of *C. jejuni* LOS antigens towards the cellular and humoral immune system induces release of C1q, macrophage activation, and production of antibodies in plasma cells. Due to molecular mimicry, these antibodies not only target LOS on *C. jejuni* but also ganglioside moieties on myelin or axons of peripheral nerves after crossing the blood nerve barrier (molecular mimicry) [31]. However, other cases, of GBS patients infected with strains of *C. jejuni* without molecular mimicry of self-antigens, exist [32]. According to the classical AIDP model, cross-reactive antigens are recognised by components of the immune system, such as macrophages and CD4+ T-cells, which help B-cells transform into plasma cells and produce neutralizing antibodies that can react with gangliosides as autoantibodies [32]. Complement activation via the classical, lectin, or alternative pathways then leads to neuronal demyelination via the membrane attack complex (MAC) (C5b-C9), inflammation caused by anaphylatoxins (C3a and C5a), and infiltration and invasion of macrophages that produce cytokines such as tumour necrosis factor-α (TNFα), as well as free radicals, such as nitric oxide [32]. Macrophages with Fc receptors and complement receptors for C3b can damage peripheral nerves also through receptor-mediated phagocytosis and induction of myelin destruction [32]. Infiltration of activated CD4+ T-cells enhances activity of macrophages through inflammatory cytokines such as interferon-γ (INF-γ) and TNF-α [32]. Alternative complement activation via lectin pathway takes place by mannose-binding lectin (MBL) [33]. Whether polymorphisms of the MBL gene contribute to susceptibility and severity of GBS is unknown, but in a study of 300 GBS patients from Bangladesh, MBL2 polymorphisms were related to reduced-serum MBL and associated with the severity of GBS [33].

Alternative Mechanisms

Another pathophysiological mechanism that explains autoimmune demyelination of peripheral axons is interaction of the central antigen-presenting cell receptor Siglec-1 with the sialylated LOS motifs found specifically on surfaces of GBS-associated *C. jejuni*. This interaction induces T-cell differentiation and autoantibody elicitation [34]. In a mouse model, autoantibody responses and associated nerve histologic changes were dependent on IL-4 production by CD4 T-cells [34].

Furthermore, nucleotide oligomerization domain (NOD) proteins, which are cytoplasmic receptors, seem to play an important role in host-innate immune responses to pathogens [35]. NODs recognise self or non-self molecules and have been implicated in many autoimmune diseases, including GBS [35]. Polymorphisms of *NOD1* genes were suspected to play a role in the pathophysiology of GBS [35]. However, in a study of 303 GBS patients, the polymorphisms in the *NOD1* and *NOD2* genes conferred no risk to susceptibility or severity of GBS [35].

Difference between Axonal and Demyelinating Lesion

Pathophysiology differs between demyelinating and axonal subtypes of GBS [2]. AMAN is primarily an antibody-mediated condition in which IgG and activated complement proteins are deposited on the nodal and inter-nodal axolemma [2]. Macrophages contribute to axonal injury by invading the periaxonal space between the axon and myelin. Antibodies can also interfere with nerve regeneration [2]. In AMAN, axonal involvement can result in axonal degeneration or rapid resolution of conduction block and abnormal nodal lengthening [2]. There is no T-cell infiltration in AMAN.

Variable Pathogenicity of *C. jejuni* Strains

Pathogenicity of *C. jejuni* is different between its various strains. The risk of GBS is estimated to be at least six times higher with strain *C. jejuni* Penner serotype HS:19 than is the average risk [36]. The reason could be that sulphated biomolecules, such as polysaccharides, can promote immune response [36]. Additionally, proteins involved in the persistence-mediated pathogenicity and restriction modification system as well as in certain methylation patterns could differentially effect gene-expression patterns of *C. jejuni* HS:19 [36].

In a study of 71 *C. jejuni* strains belonging to ST-2993, obtained from GBS patients during the large outbreak of GBS in Peru in 2019 and compared with four chicken *C. jejuni* strains via Illumina technology, *LOS* genes that were related to molecular mimicry with gangliosides in peripheral nerves were detected [24]. Phylogenetic analysis (reconstruction) showed a connection between Peruvian and Chinese GBS strains, both having *LOS* locus genes related to molecular mimicry with gangliosides in peripheral nerves [24]. In addition, ST-2993 was detected in Amazon strains recovered many years before the 2019 outbreak, but with no epidemiological connection to GBS [24]. The close relationship between human and chicken *C. jejuni* strains indicated chickens as a probable reservoir [24]. Comparative genomics revealed differences between Chinese and Peruvian strains, including the presence of a prophage inserted into the genome [24].

Why One Contracts GBS and Another Does Not

The question of why one can contract GBS from infection and another will not is unresolved. However, some speculations can be made to answer this question.

One explanation could be that coinfections are required to trigger the development of GBS. Since 6% of the GBS patients in the IGOS study showed co-infections [13], it is conceivable that a certain mixture of pathogens is required to trigger misguided autoimmunity.

A second explanation could be that the immune reaction against associated bacteria is different in each individual due to genetic differences of the immune system. In a recent study that examined 422 GBS patients for polymorphisms rs153109 and rs785575 in interleukin 27 (IL-27), the G-allele of the rs153109 polymorphism was found to be more common in GBS patients than in controls [37]. Patients with the G allele had a worse outcome than patients without this allele [37]. GBS patients had higher serum IL-27 levels than did healthy controls [37]. IL-27 levels were also higher in GBS patients with the AG/GG genotype, but those with the GG genotype had the highest IL-27 levels [37]. There is also evidence that Fc receptor polymorphisms and haplotypes are associated with GBS severity but not with susceptibility [38]. However, speculation that inborn errors of components of the immune system might explain the variable pathogenicity of *C. jejuni* has been largely ruled out.

A third explanation could be that the blood nerve barrier is more permeable in some patients than in others. However, little evidence supports this assumption. Speculations that an immunoglobulin deficiency could explain the variable pathogenicity of *C. jejuni* were excluded.

A fourth explanation could be high variability of surface antigens due to the relatively large number of hypermutable simple sequence repeat (SSR) tracts in the *C. jejuni* genome. This decreases its phenotypic stability through reversible changes in the ON or OFF expression states they are in: a phenomenon called phase variation [21]. SSRs are major drivers of phase variation in *C. jejuni* [21]. The presence of multiple SSR-mediated phase-variable genes that encode enzymes that modify surface structures, including capsular polysaccharide (CPS) and LOS, creates extreme cell surface diversity within bacterial populations, thereby promoting adaptation to selective pressures in host environments [21]. Control of SSR-mediated phase variation is important for therapeutic approaches.

A fifth explanation could be variable affinity of antibodies to gangliosides [16]. However, this speculation also remains unsupported so far. A sixth explanation could be the variable patterns of gangliosides on the surfaces of myelin cells and the axolemma.

*Mycoplasma pneumoniae*

Several cases have been reported showing that GBS can be preceded by pneumonia due to *Mycoplasma pneumoniae* [39]. The clinical presentation of GBS caused by *M. pneumoniae* does not vary from that of GBS caused by to *C. jejuni*. It has been postulated that *M. pneumoniae* triggers GBS due to bacterial production of galacto-cerebroside [40]. Treatment and outcome of *M. pneumoniae*-associated GBS are similar to treatment and outcome of GBS triggered by alternative causes.

*Haemophilus influenzae*

GBS following an infection with *Haemophilus influenzae* has been rarely reported. In most of these cases, causality was suspected but confirmed only by exclusion of alternative causes [41]. In a 24-year-old female with GBS, who required mechanical ventilation because of respiratory muscle involvement, the culture from the sputum was positive for *Haemophilus influenzae* [41]. GBS triggered by *Haemophilus influenzae* was also reported in a 65-year-old male who developed quadriparesis one week following pneumonia [42]. His recovery was complicated by depression for several months [42].

*Erlichia chaffeenensis*

GBS triggered by an infection with *Erlichia* has been reported only in one patient [43]. A 71-year-old female with fever, chills, general weakness, and dizziness for over three weeks tested positive for PCR for Erlichiosis, which was why doxycycline treatment was commenced [43]. Despite this treatment, symptoms worsened, and the patient developed numbness, areflexia, thrombocytopenia, and elevated liver enzymes [43]. She started to recover following administration of intravenous immunoglobulins (IVIGs) [43].

*Orientia tsutsugamushi*

*Orientia tsutsugamushi* is the causative agent of scrub typhus [44]. Clinical manifestations generally occur due to vasculitis and inflammation and can have variable degrees of systemic involvement [44]. Meningoencephalitis and cerebellitis are well-known neurological manifestations of scrub typhus, but occurrence of GBS is extremely rare [44]. In a 7-year-old male with typical clinical features of GBS, IgM antibodies against scrub typhus were positive and the OXK titre was high [44]. Single reports of GBS triggered by other bacteria also exist (Table 1).

Viruses

Viruses are well-known causes of GBS. Types of viruses that triggered GBS varied significantly between cohorts investigated and depended strongly on presence or absence of a virus epidemic.

Cytomegalovirus (CMV)

In a retrospective study of 86 GBS patients from Italy, 6% of patients had a concomitant infection with CMV [47]. CMV-related GBS is frequently associated with GM2 antibodies [65]. It occurs more commonly in children or adolescents than in adults and is commonly demyelinating and motor-dominant in nature [65]. Patients with CMV-related GBS can also present elevated antibodies against moesin: a protein that exists in trace amounts in the node of Ranvier [66]. These antibodies disappear during recovery from GBS. In a retrospective study of 4132 GBS patients, requirement of mechanical ventilation in 281 patients was associated with coexisting CMV infections (OR 4.83; 94% CI, 1.16–20.1) [67]. In a study of 30 paediatric GBS patients from Iran, anti-CMV IgG antibodies were detected in 97% of cases [68].

Zika Virus

During the 2013–2016 outbreak in the Americas and the Pacific, Zika virus infection also resulted in GBS [48]. In one study, Zika-associated GBS typically developed 1–2 weeks after acute infection [48]. Zika-associated GBS more commonly manifests as AIDP than as AMAN or AMSAN. In a study of 35 Zika-associated-GBS patients from French Polynesia, mean latency between onset of infection and onset of GBS was four weeks [69]. Zika infection in these patients manifested with conjunctival hyperemia (40%), cutaneous eruption (80%), fever (49%), arthralgia (63%), and distal edema of the limbs (26%) [69].

SARS-CoV-2

There is an ongoing debate as to whether SARS-CoV-2 can cause GBS, but an increasing number of patients; the temporal relation between onset of infection and onset of GBS; ruling out of alternative causes; and elevated cytokines, chemokines, and glial fibrillary acidic protein in the CSF argue in favour of a causal relation [70]. In a study 220 patients with SARS-CoV-2-related GBS, published in 95 articles as per the end of 2020, male preponderance was observed, age ranged from 8–94 years, and latency between onset of COVID-19 and GBS ranged from −10 to 90 days [49]. The GBS subtypes identified were AIDP (*n* = 118), AMAN (*n* = 13), AMSAN (*n* = 11), MFS (*n* = 7), PNC (*n* = 2), PCB (*n* = 1), and BFE (*n* = 0) [49]. SARS-CoV-2 was not detected in the CSF in any of the patients [49]. Therapy for GBS comprised IVIGs (*n* = 191), plasmapheresis (*n* = 15), steroids (*n* = 2), or no therapy (*n* = 7) [49]. Mechanical ventilation was needed for 41 patients. The outcome was assessed as complete recovery in 37 patients and as partial recovery in 119 patients, and 12 patients died [49].

Other Viruses

Dengue is not as common as a trigger of GBS as CMV or Zika. In a study of 5821 patients with dengue and from India, 3.2% were diagnosed with GBS [71]. Some of these patients developed sensory predominant GBS and responded favourably to standard treatment [50]. Even more rarely is GBS associated with a number of other viruses, including Epstein–Barr, measles, influenza-A/H1N1, hepatitis-E, enterovirus D68, alpha-viruses, chikungunya, parecho, and Toscana (Table 1).

#### 2.3.2. Vaccinations

Although GBS has been causally related to several vaccinations (Table 1), other studies do not confirm these findings [72]. During the H1N1 influenza vaccination campaign in 1976, an increased risk was estimated, at roughly one additional case of GBS for every 100,000 people who had been vaccinated [73]. However, with use of the influenza p(H1N1) vaccine, the risk of developing GBS declined to <1/1,000,000 [2]. In a study of 1295 patients with GBS, of whom 20 had received an influenza vaccination, a slightly elevated risk of GBS occurrence within one month following an influenza vaccination was calculated [74]. However, other studies did not find an increased risk of GBS from influenza vaccinations [75]. A few patients developed GBS after rabies [63,76], hepatitis-B [64], or polio vaccinations (Table 1). However, in a Chinese study of 1056 patients with GBS, no increased risk of GBS was detected for vaccination against hepatitis-B, influenza, hepatitis-A, varicella, rabies, polio (live), diphtheria, pertussis (acellular), tetanus, measles, mumps, rubella, Japanese encephalitis, or meningitis vaccines [72]. More than 400 cases of SARS-CoV-2-vaccination-related GBS have been reported as per the end of August 2022 [77]. Although some studies emphasise that overall incidence of GBS has not increased since introduction of SARS-CoV-2 vaccinations, several arguments support a causal relation [77].

#### 2.3.3. Others

Several non-infectious and non-vaccine-related triggers of GBS have been reported. In a male in his 80s, GBS was triggered by angio-immunoblastic T-cell lymphoma [78]. The pathophysiological explanation remained elusive, but it was speculated that malignancy could have triggered the immune dysregulation, that it could have been a paraneoplastic phenomenon, or that it could have been an autoimmune reaction triggered by a common exposure [78]. GBS has been also reported as a post-renal-transplant phenomenon [79]. Intravenous administration of gangliosides for trauma, surgery, stroke, or peripheral neuropathy and surgery have also been reported as rare triggers of GBS [80]. The reason why about one third of GBS patients do not have previous symptomatic infections, vaccinations, or other triggers could be due to the fact that about 30% of GI/respiratory infections become asymptomatic, according to the IGOS [13].

### 2.4. Treatment

IVIGs and plasma exchange (PE) are the mainstay treatments of GBS, especially in the first four weeks after onset of weakness [81]. According to a review of GBS, oral steroids or intravenous methylprednisolone alone is not beneficial in GBS. The combination of intravenous methyl-prednisolone and IVIGs was not superior to IVIGs alone. Similarly, PE followed by IVIGs is not significantly more effective than PE or IVIGs alone [2,81]. Clinical trials are currently underway to investigate some potential therapeutic candidates, including complement inhibitors [2]. In case GBS does not respond to established treatments, paranodopathy should be considered.

### 2.5. Outcome

Most patients with GBS respond favourably to immunotherapy, but a substantial proportion are left with disability, and death can occur [2]. According to the IGOS, nadir is reached within 2 weeks in 96% of patients and after 4 weeks in 99% of patients [2]. Cranial nerve involvement was reported in 50% of patients, autonomic dysfunction was reported in 25% of patients, and ventilator support was required in 19% of patients [2]. At nadir, 76% of patients were unable to walk independently [2]. In addition, 20 percent were unable to ambulate independently after one year, 5% died in high-income countries, and 17% died in low-income countries [2]. Other studies reported similar mortality rates. In a study of 3486 GBS patients, half of the GBS patients required admission to an ICU, 8% were intubated, 2% developed dysautonomia, and 1% died [11]. Predictors of poor outcome include advanced age, antecedent *C jejuni* infection, need for mechanical ventilation, and axonal subtype [2]. Prognostic models such as the modified Erasmus Guillain–Barré Syndrome Outcome Score (mEGOS) found decreased probability of walking independently at 6 months during week 1 after admission in patients who were older and had diarrhoea and a lower Medical Research Council sum score (MRCSS) [2]. According to the Erasmus Guillain–Barré Syndrome Respiratory Insufficiency Score (EGRIS), probability of early ventilation is increased among those with short duration of weakness from onset of symptoms, low MRCSS, and presence of facial or bulbar weakness [2].

## 3. Methods

A search of the literature in the databases PubMed, Google Scholar, and Scopus was conducted using the search terms “Guillain-Barre syndrome”, “acute, inflammatory demyelinating polyneuropathy”, “acute, motor, axonal neuropathy”, “acute, motor, sensory, axonal neuropathy”, “Miller Fisher syndrome”, and “brainstem Bickerstaff encephalitis” in combination with “infection”, “C jejuni”, “hemophilus influenzae”, “mycoplasma pneumoniae”, “cytomegalovirus”, “Epstein-Barr virus”, “measles virus”, “influenza-A virus”, “hepatitis E”, “enterovirus”, “swine influenza“, Zika”, and “SARS-CoV-2”. In addition, reference lists were searched for additional articles that matched the search criteria. Only original articles published between 1966 and September 2022 were included. Reviews, abstracts, proceedings, and editorials were excluded from the review.

## 4. Conclusions

GBS is a neuro-immunological disorder caused by an autoimmune reaction against components of the myelin membrane or the axolemma. This abnormal immune reaction is most commonly triggered by antecedent gastrointestinal or pulmonary infection; by vaccination; or by other non-infectious, non-vaccine-related conditions. The most common triggering infectious agent is bacterium *C. jejuni*. *C. jejuni* is highly pathogenic to humans, most commonly causes enteritis, and more rarely causes GBS. In about one third of GBS patients, a previous infection with *C. jejuni* can be identified. *C. jejuni* is less likely to cause GBS than CMV and causes a more severe course than other infectious agents. AIDP is caused by demyelination, whereas AMAN is antibody-mediated, attacking structures of the axolemma in the nodal or inter-nodal space. Post-infectious morbidity of human *C. jejuni* infection is strongly dependent on structure of pathogenic LOSs that trigger the innate immune system and possibly ganglioside pattern on myelin membranes and axons. Despite recent progress in clarifying pathophysiology of GBS, further advances are urgently needed to provide more effective treatments and improve outcome.

## Figures and Tables

**Table 1:** Infectious agents, vaccinations, and other triggers of GBS reported in the literature.

Trigger		Frequency	Reference
Bacteria			
	*Campylobacter jejuni*	+++	[7]
	*Mycoplamsa pneumoniae*	++	[39]
	*Hemophilus influenza*	+	[45]
	*Erlichia chaffeenensis*	+	[43]
	*Orientia tsutsugamushi*	+	[44]
	*Escherichia coli*	+	[46]
Viruses			
	Cytomegaly	+++	[47]
	Zika	+++	[48]
	SARS-CoV-2	+++	[49]
	Dengue	++	[50]
	Influenza-A (H1N1)	+	[51]
	Epstein Barr	+	[52]
	Hepatitis-E	+	[53]
	Measles	+	[54]
	Enterovirus D68	+	[55]
	Chikungunya	+	[56]
	Parecho	+	[57]
	Toscana virus	+	[58]
	Japanese encephalitis	+	[59]
Vaccinations			
	SARS-CoV-2	+++	[60]
	Influenza	+	[61]
	Polio	+	[62]
	Rabies	+	[63]
	Hepatitis-A, -B	+	[64]
Others			

+++: highly prevalent, ++: moderate prevalence, +: rare.

## Data Availability

All data reported are available from the corresponding author.
